# Intraosseous Varix of the Left Tibia Causing Severe Lower Extremity Pain: A Report of a Rare Case

**DOI:** 10.7759/cureus.107838

**Published:** 2026-04-27

**Authors:** Muhammad Usman, Arsalan Ahmed, Nabahat Shafi, Muhammad Usman Shahbaz, Muhammad Zawar Asif

**Affiliations:** 1 Internal Medicine, Mercy Hospital Fort Smith, Fort Smith, USA; 2 Internal Medicine, Arkansas College of Osteopathic Medicine, Fort Smith, USA; 3 Medicine, Liaquat University of Medical and Health Sciences, Karachi, PAK; 4 Medicine, Dow University of Health Sciences, Civil Hospital Karachi, Karachi, PAK

**Keywords:** musculoskeletal radiology, rare clinical presentation, unexplained leg pain, varicose veins of the lower extremity, vascular anomaly, vascular sclerotherapy

## Abstract

Varicose veins are venous anomalies of the peripheral venous system that commonly present with lower extremity pain and swelling. Intraosseous varices are a very rare subtype of varicose veins with an atypical presentation and thus pose a significant diagnostic challenge, leading to prolonged patient morbidity. Only a handful of cases have been reported of this rare entity.

We report a case of a 73-year-old male with a history of hyperlipidemia who presented with a 4-week history of worsening left lower extremity pain. Initial workup, including labs, radiography, and ultrasound Doppler, ruled out fracture, electrolyte imbalances, deep venous thrombosis, myopathies, and rhabdomyolysis. A subtle tibial shadow on radiography prompted MRI evaluation. It demonstrated a well-defined intramedullary lesion within the tibia exhibiting high signal intensity on T2-weighted sequences, low-to-intermediate signal on T1-weighted images, and avid post-gadolinium enhancement--findings consistent with a prominent tibial intraosseous varix. The patient was initially managed conservatively with compression stockings and daily exercise with minimal relief over the next eight weeks. The patient therefore underwent sclerotherapy of the left tibial varicose vein, leading to significant symptomatic improvement at the four-week follow-up.

This case highlights the significance of rare and atypical clinical entities, such as intraosseous varicose veins of long bones, which pose considerable diagnostic challenges due to persistent symptoms and benign initial workup. It also underscores the critical role of advanced imaging like MRI in establishing the diagnosis and helps distinguish it from aggressive osseous lesions. Although a conservative approach may provide some benefit initially, interventional therapies like sclerotherapy should be considered in cases where symptoms persist despite conservative measures.

## Introduction

Varicose veins are a vascular anomaly commonly involving the lower limbs, characterized by tortuous, distorted veins [1]. It commonly affects 10-40% of the population between the ages of 30 and 70 years [2,3]. Common symptoms are lower limb pain and pruritus, leading to severe complications like thrombosis, hemorrhage, and ulceration [3]. They typically involve the superficial venous system with important risk factors including advancing age, Caucasian race, and multiple gestations. Although it affects the superficial veins, intraosseous involvement is extremely rare [4,5]. Rezaie ES et al. reported that only 47 patients with intraosseous venous drainage of varicose veins have been described in the literature [3].

Intraosseous venous anomalies cause increased pressure in the venous system, with affected individuals presenting with chronic pain and lower limb edema [4]. The exact pathogenesis is not completely understood. Some evidence suggests that it can be due to prior venous insufficiency, defects within the endothelial cells, and valvular dysfunction [3]. The atypical presentation of the disease and the limited number of reported cases further create diagnostic delays [5]. 

This report describes a case of a 73-year-old male patient who presented with the complaint of lower extremity pain for 4 weeks, with no prior history of venous insufficiency.

## Case presentation

A 73-year-old male with a past medical history significant for hyperlipidemia presented to the emergency department with complaints of left lower extremity pain, ongoing for the past 4 weeks. The patient reported his pain to be of sudden onset about 4 weeks ago, located in the midshaft of the left tibia without any radiation, worsening with walking. The pain has progressively worsened in each subsequent episode to the point that he is unable to walk without a walker. The patient reported that his pain lasted around three to four hours and then resolved on its own. He was evaluated recently at a local clinic, and fracture as well as deep vein thrombosis (DVT) were ruled out after normal results of ultrasound Doppler of the left lower extremity and radiographic imaging. He reported that he tried gabapentin without any significant relief in his pain. He also reported that he has been taking rosuvastatin for the past 20 years, the dose of which was recently increased to 20 mg daily; however, his symptoms had been worsening prior to his dose increase. Physical examination revealed 1+ left lower extremity edema, mild tenderness to palpation over the left anteromedial muscle compartment with no mass or crepitus noted, no visible dilated veins, erythema, muscle wasting, or skin rash was present, intact distal pulses bilaterally, and no limitation to range of motion on either of the lower extremities.

Lab work, including complete blood count, erythrocyte sedimentation rate, C-reactive protein, serum electrolytes, and CK levels, was normal.

**Figure 1:**
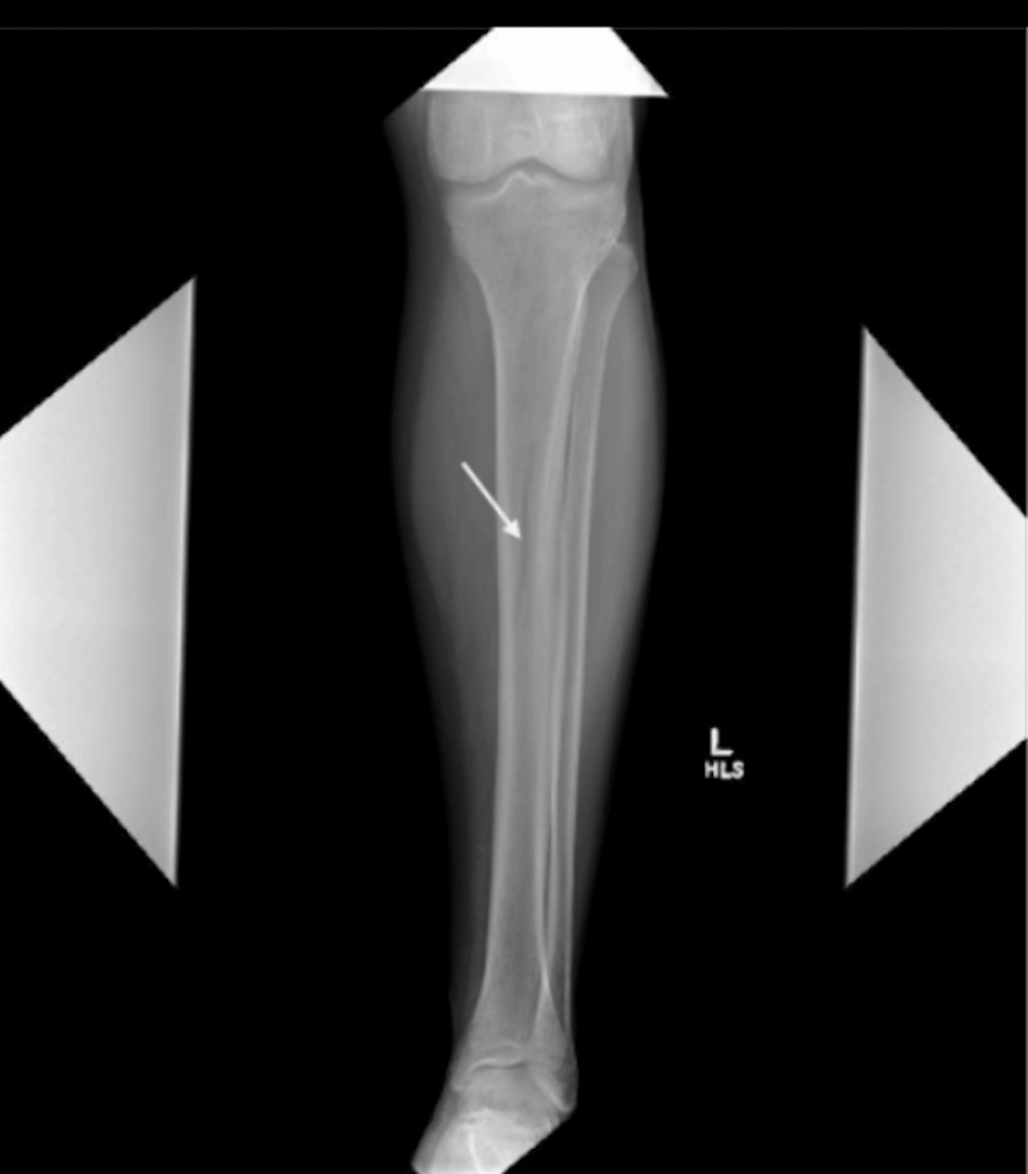
X-ray of the left tibia showing a subtle shadow in the mid tibial shaft; no fractures are present

An X-ray of the left tibia and fibula was negative for any fracture; however, it did show a shadow through the tibial shaft (Figure 1).

**Figure 2:**
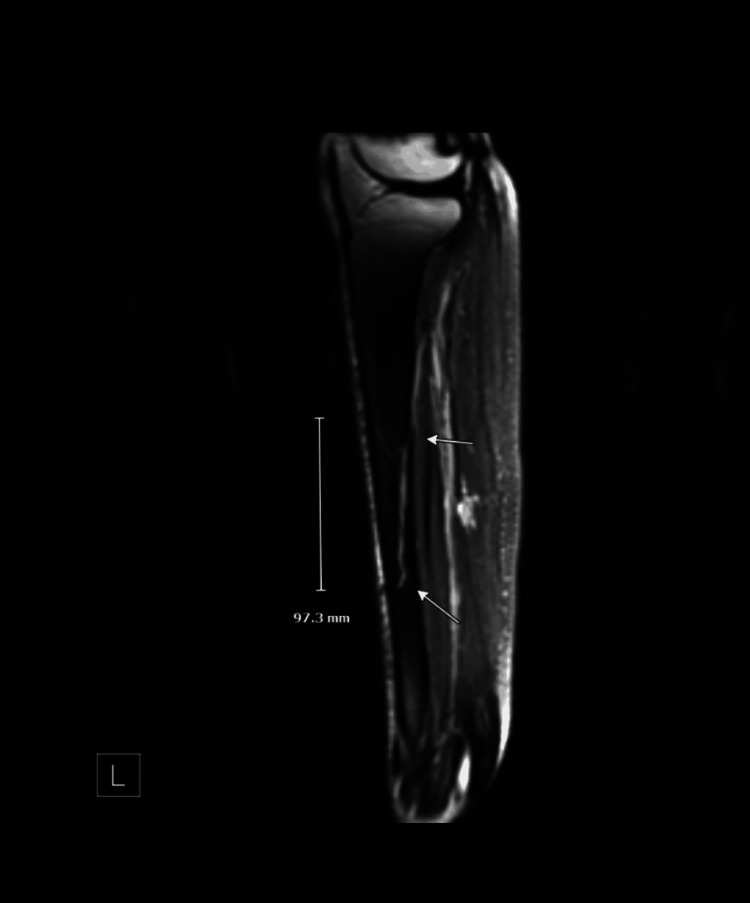
MRI of the left tibia and fibula, T2 sagittal view, revealing intraosseous varix in the central portion of the tibial diaphysis, with a craniocaudal extent of almost 10 cm

**Figure 3:**
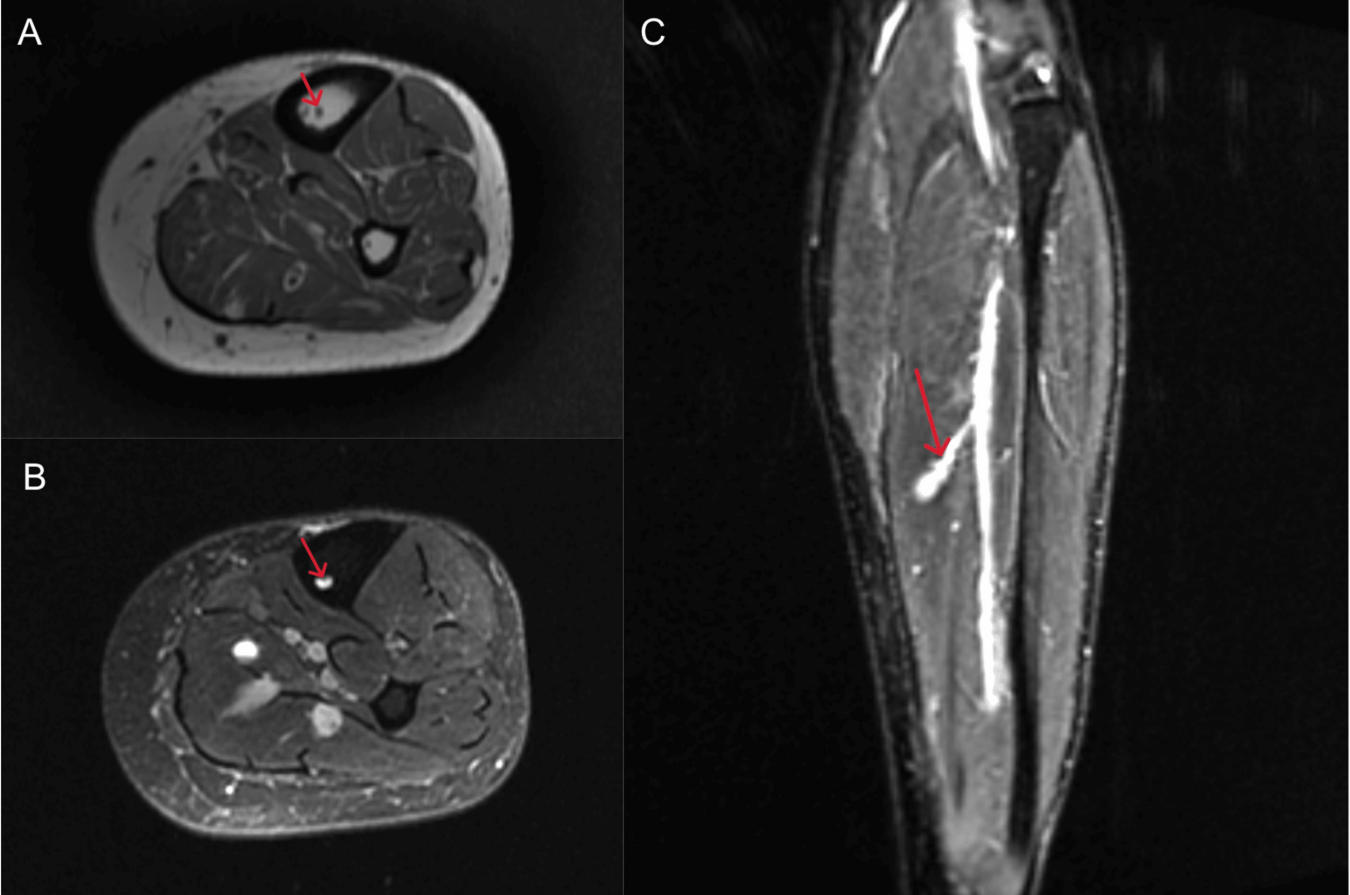
MRI of the left tibia and fibula (panel A: axial T1 fat-saturated sequence, panel B: axial T2 fat-saturated sequence, and panel C: coronal short tau inversion recovery sequence) showing a prominent intraosseous varix at the mid tibial level

A subsequent MRI of the left tibia and fibula without contrast revealed a well-defined intramedullary lesion within the tibia, which enters the posterior diaphysis in the proximal third of the bone and traverses down to the distal third before exiting anteriorly out of the anterior tibial cortex, with a total craniocaudal extent of approximately 10 cm, as well as evidence of mild venous insufficiency with varicosities in the distal soleus muscle. It exhibits high signal intensity on T2-weighted and short tau inversion recovery (STIR) sequences, low-to-intermediate signal on T1-weighted images, and avid post-gadolinium enhancement--findings consistent with a prominent tibial intraosseous varix (Figures 2-3).

In addition, normal radiographic images of the left lower extremity, the patient's pain pattern, and benign physical examination further supported the MRI evidence of intraosseous varix as the likely etiology of the patient's current presentation. 

Bilateral lower extremity arterial duplex was performed with no evidence of lower extremity arterial stenosis. Bilateral lower extremity venous reflux duplex revealed a short segment of right greater saphenous vein reflux in the calf, and left common femoral vein reflux without any significant reflux in the left greater saphenous vein. After a multidisciplinary discussion, a shared decision was made for conservative therapy with daily exercise and compression stockings, as the patient preferred to avoid invasive procedures unless necessary. He initially had some improvement of his symptoms with conservative management; however, he continued to have intermittent episodes of intense pain over the next 6 to 8 weeks, with pain intensity staying 10/10 during all these episodes. At that point, a decision was made to proceed with sclerotherapy of the left soleus/tibial varicose vein. 

He was advised to continue daily exercise and avoid prolonged standing whenever possible. At the four-week follow-up, the patient reported significant improvement in his pain and quality of life.

## Discussion

Intraosseous vascular anomalies are rare entities that may be underdiagnosed due to their variable and nonspecific clinical and radiographic presentation [6,7]. Intraosseous varix, a subtype of venous malformation characterized by dilated venous channels within the medullary cavity, is particularly uncommon and most often reported in the vertebral column and craniofacial skeleton, with very limited cases involving long bones such as the tibia [4,6]. This unusual location, as seen in our patient, contributes significantly to diagnostic uncertainty and to the sense of novelty.

The clinical presentation of intraosseous varices is nonspecific and often mimics more common musculoskeletal, vascular, or neuropathic conditions [4,8]. Patients may present with chronic or intermittent localized pain, often exacerbated by weight-bearing or limb movement, as observed in our case [6]. Because of non-specific symptoms, initial differential diagnoses are broad and usually include deep venous thrombosis, cellulitis, osteomyelitis, statin-induced myopathy, peripheral neuropathy, primary bone tumors, metastasis, or venous malformation. Due to overlapping imaging features with other osseous lesions, such as fibrous dysplasia, intraosseous meningioma, metastasis, and primary bone tumors, the diagnosis may be delayed or overlooked [5]. In our patient, repeated evaluations for thrombotic and infectious etiologies were negative, highlighting the diagnostic challenge and tendency for delayed recognition.

Radiographically, intraosseous vascular lesions may be subtle or entirely occult on plain radiographs [9], as demonstrated in this case, where only a vague tibial shaft shadow was initially appreciated. MRI plays an important role in evaluating the lesion and helps identify intraosseous vascular channels and in excluding associated soft-tissue or osseous masses [10]. In our patient, MRI confirmed the presence of a prominent intraosseous varix without evidence of tumor, infection, or inflammatory process. These findings underscore the complementary role of multimodal imaging in establishing a definitive diagnosis.

The pathophysiology of intraosseous venous malformations is considered to represent a congenital vascular malformation characterized by abnormal development of venous channels within bone, leading to dilated, low-flow vascular spaces. These lesions demonstrate progressive expansion due to venous ectasia rather than neoplastic endothelial proliferation. However, the exact underlying mechanisms remain incompletely understood, largely due to the rarity of reported cases. In general, these lesions are benign and slow-growing, and clinical symptoms typically arise as a result of lesion enlargement with cortical thinning, osseous expansion, or mass effect on adjacent structures [9,11], potentially explaining the severe pain experienced by our patient. The intermittent episodes of pain are attributed to venous hypertension within the non-distensible bony compartment, with symptoms resolving after a few hours when venous drainage normalizes. This phenomenon is also responsible for marrow edema and periosteal reaction in the long term [2,3].

Management strategies for intraosseous venous malformations are not standardized and are guided by symptom severity and anatomical involvement. Conservative measures are preferred in uncomplicated cases, while image-guided interventions and surgical procedures may be required in patients with persistent symptoms, failed embolization/sclerotherapy, or structural bone compromise such as cortical destruction or fracture [12,13]. In the present case, conservative management with compression stockings and mobilization resulted in symptomatic improvement, supporting the non-aggressive nature of the lesion [14].

This case is notable due to the rare involvement of the tibia, the severity of pain leading to functional impairment, and the initial extensive workup excluding more common etiologies such as thrombosis, infection, and statin-induced myopathy. It highlights the importance of maintaining a broad differential diagnosis in patients with unexplained limb pain and emphasizes the role of advanced imaging in identifying uncommon vascular bone lesions. Due to the rarity of this entity, with some lesions being asymptomatic, having limited long-term follow-up data [3], and management protocols not being well-standardized, this remains an area of active research.

## Conclusions

Intraosseous varix of the tibia is an exceptionally rare vascular anomaly that may present with nonspecific limb pain and mimic more common musculoskeletal conditions. This case emphasizes the importance of considering vascular osseous lesions in persistent unexplained limb pain and highlights the critical role of MRI in establishing diagnosis and guiding conservative management. This case further highlights the role of definitive treatments like vascular sclerotherapy when initial conservative measures fail to provide symptom relief.
